# Effectiveness and Safety of Cystic Fibrosis Transmembrane Conductance Regulator Modulators in Children With Cystic Fibrosis: A Meta-Analysis

**DOI:** 10.3389/fped.2022.937250

**Published:** 2022-06-29

**Authors:** Qiyu Li, Siyuan Liu, Xuemei Ma, Jiaping Yu

**Affiliations:** Department of Pediatrics, General Hospital of Northern Theater Command, Shenyang, China

**Keywords:** CFTR (cystic fibrosis transmembrane conductance regulator), cystic fibrosis – CF, corrector, potentiator, cystic fibrosis transmembrane conductance regulator

## Abstract

**Background and Aim:**

Cystic fibrosis (CF) is a genetic disease that is difficult to treat and caused by dysfunction of the cystic fibrosis transmembrane conductance regulator (CFTR) protein. Small molecules have been used to treat the symptom caused by CFTR mutations by restoring CFTR protein function. However, the data on children with CF are scarce. This meta-analysis aimed to evaluate the effectiveness and safety of this therapy in children diagnosed with CF.

**Materials and Methods:**

Relevant studies were identified through searching medical databases before April 1, 2022. The primary outcomes of ppFEV_1_, lung clearance index_2.5_ (LCI_2.5_), sweat chloride concentration (SwCI), and Cystic Fibrosis Questionnaire-Revised (CFQ-R) score were pooled and analyzed. The secondary outcomes were nutritional status (weight, BMI, stature, and their z-score) and adverse events under therapy.

**Results:**

A total of twelve studies were included. Compared with the placebo group, the pooled outcome of the ppFEV1, LCI_2.5_, SwCI, and CFQ-R score were improved by 7.91 {[95% confidence interval (CI), 3.71–12.12], –1.00 (95% CI, –1.38 to –0.63), –35.22 (95% CI, –55.51 to –14.92), and 4.45 (95% CI, 2.31–6.59), respectively}. Compared with the placebo group, the pooled result of the change in weight was improved by 1.53 (95% CI, 0.42–2.63). All the aforementioned results were also improved in single-arm studies. No clear differences in adverse events were found between CFTR modulator therapy and the placebo group.

**Conclusion:**

CFTR modulators could improve multiaspect function in children with CF and result in comparable adverse events.

## Introduction

Cystic fibrosis (CF) is a life-shortening genetic disease characterized by a progressive decrease in lung function, pulmonary exacerbations, poor nutritional status, and eventually premature death (1, 2). The fundamental reason for CF is mutations in cystic fibrosis transmembrane conductance regulator (CFTR) genes coding the CFTR protein, which is located at the apical membrane of epithelial cells (3). The defective CFTR protein causes impairment in transporting chloride anions at epithelial surfaces, which induces the corresponding loss of function in multiple organs, including lungs, pancreas, and gastrointestinal tracts (1).

More than 2000 genetic variants have been found, which are classified into six classes (Class I to Class VI) according to their molecular mechanisms of dysfunction (4). The p.Phe508del CFTR mutation, which is found in 90% of the population, causes trafficking defects (Class II) (4). The Gly551Asp mutation (also known as G551D), which is the most common Class III, or gating mutation, is present in 4% of people with CF (PwCF) in the United States, diminishing the opening probability of the channel (4, 5). Nearly 50% of patients have homozygous p.Phe508del CFTR mutations (F/F), and almost 33% have heterozygous minimal-function CFTR mutations, which is with one mutation that is minimal function and one F508del. (F/MF) (6). Classes IV–VI cause limited CFTR dysfunction, which is called residual function (RF) mutation. Most patients with these RF (F/RF) or gating (F-gating) CFTR mutations are heterozygous for the p.Phe508del mutation (6).

According to the CFTR mutation, small molecules have been developed to restore CFTR protein function (4). Generally, the modulators can be classified as CFTR potentiators (e.g., ivacaftor, IVA) and correctors. The correctors are divided into first-generation CFTR correctors (e.g., lumacaftor, LUM and tezacaftor, TEZ) and next-generation correctors (elexacaftor, ELX). IVA was the first of these molecules that proved effective in phase III clinical trials (7), targeting F-gating CFTR mutations, with significant improvement in lung function and nutrition status (4). The following clinical trials, including dual combination therapy (LUM/IVA and TEZ/IVA) (8, 9) and triple combination therapy (ELX/TEZ/IVA) (10, 11), manifested that these molecules improved pulmonary function, CFTR function, and quality of life significantly.

However, the patients in most of these studies were adults or adolescents (older than 12 years). The safety and effectiveness of small molecules in children with CF are relatively less known. The potential reason may be associated with the risk of adverse events and uncertain efficacy compared with the favorable results in adults. Nevertheless, early treatment before any severe functional deficiency may be an optimal strategy for patients with CF. Hence, this meta-analysis was conducted to examine current studies on these small molecules in children with CF in terms of efficacy and safety, according to different mutation genotypes.

## Materials and Methods

### Study Search

This meta-analysis was performed according to the Preferred Reporting Items for Systematic Reviews and Meta-analyses guidelines (12). The checklist is presented in Supplementary Table 1. The literature search was performed through PubMed, Web of Science, and Cochrane Library on April 1, 2022. The search terms and queries are presented in Supplementary Table 2.

### Study Selection and Eligibility Criteria

Relevant studies were collected, and duplicates were removed (identification). The studies relevant to our analysis were selected for full-text review based on the titles and abstracts (screening). Studies were screened according to the inclusion and exclusion criteria. An additional search was performed on the references of the included studies to further identify potentially eligible studies.

The inclusion criteria were as follows: (1) population: children (≤ 11 years old) diagnosed with CF having at least one CFTR mutation; (2) intervention: patients who received monotherapy, dual combination therapy or triple combination therapy for CF; (3) comparison: patients who received placebo treatment; (4) outcomes: primary outcomes included the absolute change from baseline in predicted forced expiratory volume in 1 s (ppFEV_1_), absolute change from baseline in lung clearance index_2.5_ (LCI_2.5_), absolute change from baseline in sweat chloride concentration (SwCI), and absolute change from baseline in Cystic Fibrosis Questionnaire-Revised (CFQ-R) respiratory domain score. The secondary outcomes included adverse events, nutrition parameters (weight, BMI, and stature), pancreatic exocrine function (fecal elastase 1, FE1, and immunoreactive trypsinogen concentration, IRT); and (5) study design: single-arm study or randomized controlled trials (RCTs).

The exclusion criteria were as follows: (1) case reports, abstracts, or reviews, (2) studies with mixed data, including adolescents or adults, (3) no reporting of outcomes of interest, (4) studies published in languages other than English, and (5) preclinical studies or experiments *in vitro*.

### Data Collection

A formalized table was independently used by Q.Y.L and J.P.Y to extract data from each study. The following information was included: (1) authors, (2) publication year, (3) study design, (4) setting (single center/multicenter); (5) enrollment period, (6) number of patients, (7) components of therapy, (8) components of active control therapy, (9) absolute change in ppFEV_1_ and LCI_2.5_, (10) absolute change in SwCI, (11) absolute change in CFQ-R score, (12) any adverse events, (13) mutation type, (14) absolute change in weight, weight-for-age z-score, BMI, BMI-for-age z-score, stature, and stature-for-age z-score, and (15) absolute change in FE1 and IRT.

### Statistical Analysis

Our meta-analysis included two types of data. One included continuous variables from single-arm studies, and the pooled single proportion rates were analyzed using Stata software, version 12.0 (2011; Stata Corp., TX, United States). The other included continuous variables from RCTs. RevMan 5.3 (Cochrane) was used for statistical analysis. The inverse variance method and mean difference (MD) were used. All pooled results were calculated with a random-effects model because it provided more conservative estimates. All statistical values were reported with 95% confidence intervals (CI). The subgroup analysis was conducted by drug and genotype to diminish the heterogeneity as much as possible.

## Results

### Search Results

**FIGURE 1:**
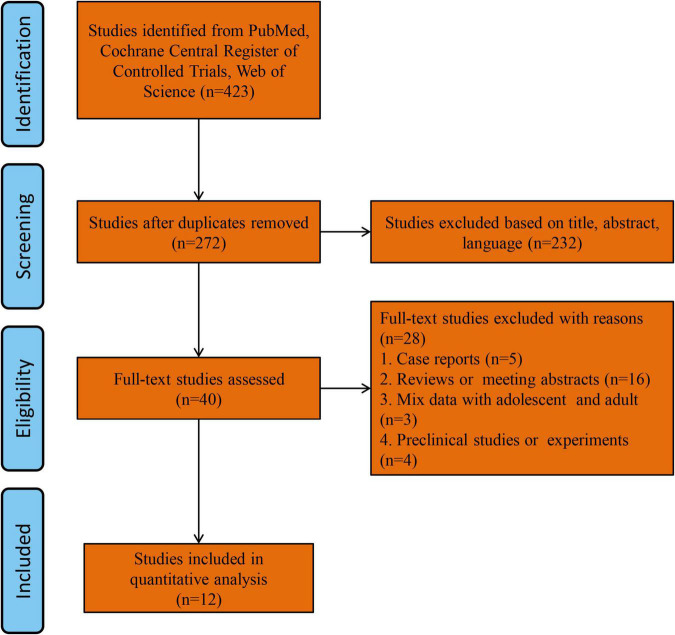
Flow chart of this meta-analysis.

The study flowchart is shown in Figure 1. Forty records were eligible for full-text review. Five case series or report studies were excluded. Sixteen studies were excluded for being reviews or meeting abstracts. Three studies were excluded for containing mixed data, including adolescents and adults, and four studies were excluded for being preclinical studies or experiments. Finally, 12 studies were included in the final analysis (13–24).

### Main Characteristics of the Included Studies

Nine of the included studies were single-arm studies (13–21), and three were RCTs (22–24). The main characteristics of the included studies are shown in Table 1. All the included studies were multicenter phase 3 trials. Seven of 12 studies adopted a two-part study design to examine the initial safety in part A for a short period, and then conduct the rest of the trial in part B. The main information on patients and treatment are shown in Table 2. The most common length of treatment duration was 24 weeks (13–16, 18–20, 22, 23), including two long-term studies from Rosenfeld et al. (108 *weeks) (17) and Sawicki et al. (96 weeks) (21), which were the extension of three previous studies (13, 18, 24). The ages of children in the included studies were 4–12 months (19), 12–24 months (15), 2–5 years (13, 16, 17), and 6–11 years (14, 18, 20–24). The drug doses were mostly administered according to the weight of the patients and mutation types. All the CFTR gating mutations were given by IVA (monotherapy) (13, 15, 17, 19, 22). Patients with only F/F mutation were treated with LUM + IVA (dual combination therapy) (14, 16, 23), and patients with F/F or F/RF mutation were treated with TEZ + IVA (dual combination therapy) (18, 21, 24). Only one triple combination therapy (ELX + TEZ + IVA) was used in patients with F/F or F/MF mutation (20).

**TABLE 1:** The main characteristics of included studies.

References	Trial name	Country	Setting	Phase	Two part study
**Single-arm**					
Davies et al. (13)	KIWI	United Kingdom	Multicenter	Phase 3	Yes
Milla et al. (14)	VX13-809-011 Part B	United States	Multicenter	Phase 3	No
Rosenfeld et al. (15)	ARRIVAL	United States	Multicenter	Phase 3	Yes
McNamara et al. (16)	VX15-809-115	United States	Multicenter	Phase 3	Yes
Rosenfeld et al. (17)	KLIMB	United States	Multicenter	Phase 3	No
Walker et al. (18)	VX15-661-113	United States	Multicenter	Phase 3	Yes
Davies et al. (19)	ARRIVAL	United Kingdom	Multicenter	Phase 3	Yes
Zemanick et al. (20)	VX18-445-106	United States	Multicenter	Phase 3	Yes
Sawicki et al. (21)	VX17-661-116	United States	Multicenter	Phase 3	Yes
**RCT**					
Davies et al. (22)	ENVISION	United Kingdom	Multicenter	Phase 3	NO
Ratjen et al. (23)	VX14-809-109	Canada	Multicenter	Phase 3	NO
Davies et al. (24)	VX16-661-115	United Kingdom	Multicenter	Phase 3	NO

**TABLE 2:** The main information of patients and treatment.

Author	Treatment duration (weeks)	Age	Drug dose	Patients (n)	Mutation
Davies	24	2–5 y	Weight < 14kg, IVA(50mg/12h); Weight > 14kg, IVA(75mg/12h)	*n* = 10 (50mg/12h) *n* = 24 (75mg/12h)	CFTR gating
Milla	24	6–11 y	LUM (200mg/12h) + IVA(250mg/12h)	*n* = 58	F/F
Rosenfeld	24	12–24 m	7 < Weight < 14kg, IVA(50mg/12h); 14 ≤ Weight < 25kg, IVA(75mg/12h)	*n* = 19	CFTR gating
McNamara	24	2–5 y	Weight < 14kg, LUM (100mg/12h) + IVA(125mg/12h); Weight > 14kg, LUM (150mg/12h) + IVA(188mg/12h)	*n* = 60	F/F
Rosenfeld	108	2–5 y	Weight < 14kg, IVA(50mg/12h); Weight > 14kg, IVA(75mg/12h)	*n* = 33	CFTR gating
Walker	24	6–11 y	Weight < 40kg, TEZ (50mg/QD) + IVA(75mg/12h); Weight > 40kg, TEZ(100mg/12h) + IVA(150mg/12h)	*n* = 70	F/F or F/RF
Davies	24	4–12 m	Weight < 7kg,IVA(25mg/12h); 7kg < Weight < 14kg,IVA(50mg/12h)	*n* = 17	CFTR gating
Zemanick	24	6–11 y	Weight < 30kg, ELX(100mg/QD) + TEZ (50mg/QD) + IVA(75mg/12h); Weight ≥ 30kg, ELX(200mg/QD) + TEZ(100mg/12h) + IVA(150mg/12h)	*n* = 64	F/F or F/MF
Sawicki	Study 661-115: 8 + 96; Study 661-113B: 24 + 96	6–11 y	Weight < 40kg,TEZ (50mg/QD) + IVA(75mg/12h); Weight > 40kg,TEZ(100mg/12h) + IVA(150mg/12h)	*n* = 130	F/F or F/RF
Davies	24 and 48	6–11 y	IVA(150mg/12h)	IVA: *n* = 26 Placebo: *n* = 26	CFTR gating
Ratjen	24	6–11 y	LUM(200mg/12h) + IVA(250mg/12h)	LUM + IVA: *n* = 103 Placebo: *n* = 101	F/F
Davies	8	6–11 y	Weight < 40kg, TEZ(50mg/QD) + IVA(75mg/12h); Weight ≥ 40kg, TEZ(100mg/QD) + IVA(150mg/12h)	TEZ + IVA: *n* = 54 Placebo: *n* = 10	F/F or F/RF

### Pooled Analysis of Lung Function (ppFEV_1_ and LCI_2.5_)

**FIGURE 2:**
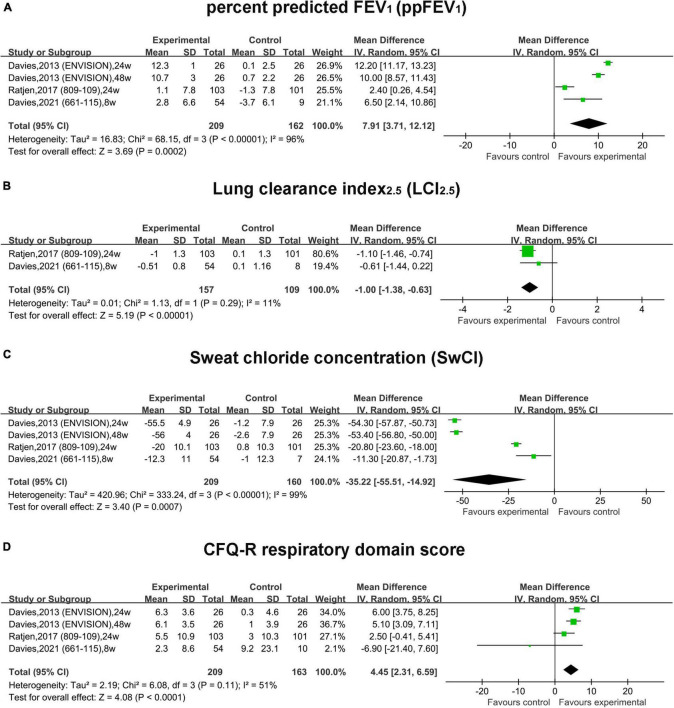
Forest plots of the RCT studies evaluating the efficacy of therapy vs. placebo. **(A)** ppFEV1. **(B)** LCI_2.5_. **(C)** Sweat chloride concentration. **(D)** CFQ-R respiratory domain score.

In RCTs, the pooled result of ppFEV_1_ in the treatment group was much more improved than that in the placebo group (MD, 7.91; 95% CI, 3.71 to 12.12) with significant heterogeneity (*I*^2^ = 96%) (Figure 2A). Similarly, the pooled effect of LCI_2.5_ was also improved in the treatment group compared with that in the placebo group (MD, –1.00; 95% CI, –1.38 to –0.63) with small heterogeneity (*I*^2^ = 11%) (Figure 2B).

**FIGURE 3:**
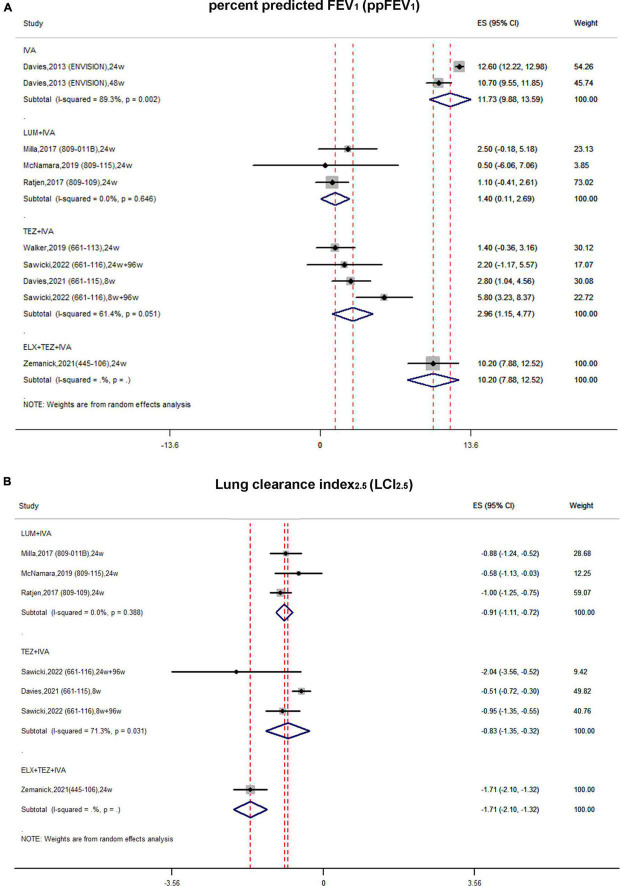
Forest plots of the single-arm studies evaluating the effectiveness of **(A)** ppFEV1 and **(B)** LCI_2.5_.

In single-arm studies, the pooled estimate of the absolute change in ppFEV_1_ of the IVA subgroup was improved by 11.73 (95% CI, 9.88–13.59) with significant heterogeneity (*I*^2^ = 89.3%) (Figure 3A). In the LUM + IVA subgroup and TEZ + IVA subgroup, the pooled estimate of ppFEV_1_ was elevated by 1.4 (95% CI, 0.11–2.69) and 2.96 (95% CI, 1.15–4.77), respectively (Figure 3A). The heterogeneity of these two subgroups was relatively non-significant (Figure 3A). The triple combination could increase ppFEV_1_ by 10.2 (95% CI, 7.88–12.52) (Figure 3A).

In single-arm studies, the pooled estimate of the absolute change in LCI_2.5_ in the LUM + IVA subgroup was improved by –0.91 (95% CI, –1.11 to –0.72) with no obvious heterogeneity (*I*^2^ = 0%) (Figure 3B). Similarly, the pooled estimate of LCI_2.5_ in the TEZ + IVA group was improved up to –0.83 (95% CI, –1.35 to –0.32) with significant heterogeneity (Figure 3B). The triple combination improved LCI_2.5_ by –1.71 (95% CI, –2.1 to –1.32) (Figure 3B).

### Pooled Analysis of Cystic Fibrosis Transmembrane Conductance Regulator Function Sweat Chloride Concentration and Life Quality (Cystic Fibrosis Questionnaire-Revised Score)

In RCTs, the pooled absolute change of SwCI in the treatment group was improved by –35.22 mmol/L (95% CI, –55.51 to –14.92) compared with the placebo group with significant heterogeneity (*I*^2^ = 96%) (Figure 2C). The pooled estimate of the CFQ-R score was increased by 4.45 (95% CI, 2.31–6.59) in the treatment group compared with the placebo group with non-significant heterogeneity (*I*^2^ = 51%, *P* = 0.11) (Figure 2D).

**FIGURE 4:**
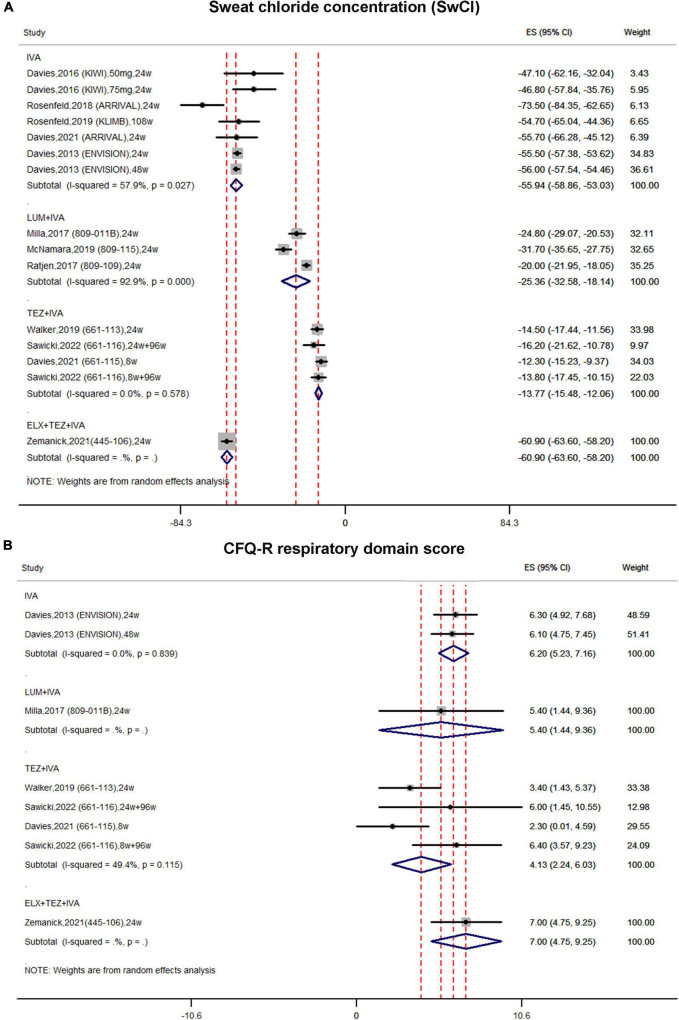
Forest plots of the single-arm studies evaluating the effectiveness of **(A)** sweat chloride concentration and **(B)** CFQ-R respiratory domain score.

In single-arm studies, the pooled estimate of the absolute change in SwCI in the IVA subgroup was improved by –55.94 mmol/L (95% CI, –58.86 to –53.03) with moderate heterogeneity (*I*^2^ = 57.9%) (Figure 4A). In the LUM + IVA subgroup, the pooled result of SwCI was improved by –25.36 mmol/L (95% CI, –32.58 to –18.14) with significant heterogeneity (*I*^2^ = 92.9%) (Figure 4A). In the TEZ + IVA group, the pooled estimate of SwCI was improved by –13.77 mmol/L (95% CI, –15.48 to –12.06) with non-significant heterogeneity (*I*^2^ = 0%) (Figure 4A). In the triple combination subgroup, the pooled estimate of SwCI was significantly improved by –60.9 mmol/L (95% CI, –63.6 to –58.2) (Figure 4A).

In single-arm studies, the pooled estimate of the CFQ-R score in the IVA subgroup was increased by 6.2 (95% CI, 5.23–7.16) (Figure 4B). Similarly, the pooled estimate of the CFQ-R score was also improved in both the LUM + IVA subgroup and the TEZ + IVA subgroup by 5.4 (95% CI, 1.44–9.36) and 4.13 (95% CI, 2.24–6.03), respectively (Figure 4B). In the triple combination group, the pooled result of the CFQ-R score was also improved by 7.00 (95% CI, 4.75–9.25) (Figure 4B).

### Pooled Analysis of the Nutritional Status (Weight, BMI, and Stature)

**FIGURE 5:**
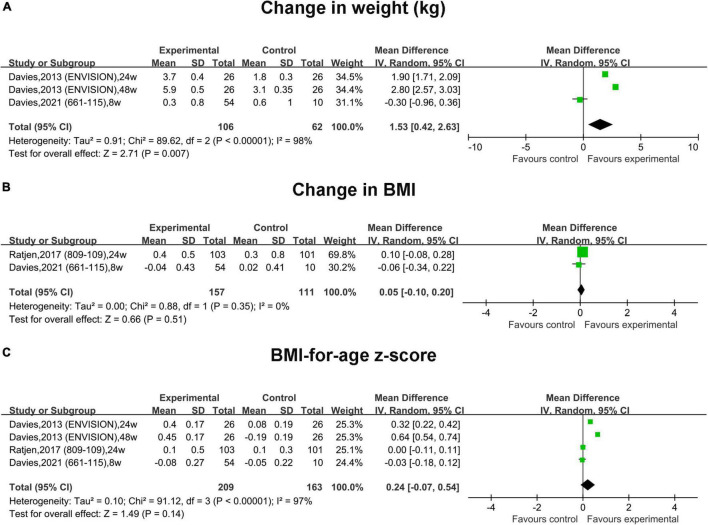
Forest plots of the RCT studies evaluating the efficacy of therapy vs. placebo. **(A)** Change in weight. **(B)** Change in BMI. **(C)** BMI-for-age z-score.

In RCTs, the pooled change in weight in the treatment group was slightly increased by 1.53 kg (95% CI, 0.42–2.63) compared with that in the placebo group, and the heterogeneity was significant (*I*^2^ = 98%) (Figure 5A). The pooled change in the BMI and BMI-for-age z-score was slightly increased compared with that in the placebo group by 0.05 (95% CI, –0.10 to 0.20) and 0.24 (95% CI, –0.07 to 0.54), respectively. However, no statistical significance was observed (Figures 5B,C).

In single-arm studies, the pooled change in weight in the IVA subgroup was increased by 4.80 kg (95% CI, 2.64–6.96) (Supplementary Figure 1A). The pooled estimates in the LUM + IVA subgroup and the TEZ + IVA subgroup were also improved by 1.40 kg (95% CI, 1.17–1.63) and 4.62 kg (95% CI, 1.96–7.28), respectively (Supplementary Figure 1A). The pooled change in the weight-for-age z-score in the IVA subgroup and the LUM + IVA group was increased by 0.27 (95% CI, 0.06–0.49) and 0.26 (95% CI, 0.15–0.37), respectively (Supplementary Figure 1B). However, the pooled change in the weight-for-age z-score in the TEZ + IVA group was basically not changed (95% CI, –0.06 to 0.05) (Supplementary Figure 1B). The pooled change in the BMI in the LUM + IVA subgroup was improved by 0.44 (95% CI, 0.26–0.62) (Supplementary Figure 2A). Although the pooled change in the BMI in the TEZ + IVA subgroup was also improved by 0.83, no statistically significant difference was noted (95% CI, –0.09 to 1.75) (Supplementary Figure 2A). As for the BMI-for-age z-score, the pooled results in the IVA subgroup and the LUM + IVA group was improved by 0.37 (95% CI, 0.29–0.45) and 0.16 (95% CI, 0.08–0.25), respectively (Supplementary Figure 2B). The pooled estimates in the TEZ + IVA subgroup were slightly decreased by –0.04 (95% CI, –0.09 to 0.00); however, no statistically significant difference was found (Supplementary Figure 2B).

As for the change in stature, the pooled result in the IVA subgroup and the TEZ + IVA subgroup was improved by 3.6 cm (95% CI, 3.29–3.91) and 7.18 cm (95% CI, 3.91–10.45), respectively (Supplementary Figure 3A). The pooled outcome in the stature-for-age z-score in the IVA subgroup was increased by 0.13 (95% CI, 0.00–0.27) (Supplementary Figure 3B); however, the statistically significant difference was not significant. In the LUM + IVA subgroup and the TEZ + IVA subgroup, the pooled estimate of the stature-for-age z-score was slightly improved by 0.09 (95% CI, 0.03–0.15) and 0.02 (95% CI, –0.02 to 0.06), respectively (Supplementary Figure 3B).

### Pooled Analysis of Pancreatic Exocrine Function (Fecal Elastase 1 and Immunoreactive Trypsinogen Concentration)

The pooled estimate of FE1 in the IVA subgroup was 129.09 μg/g (95% CI, 95.06–163.11) with non-significant heterogeneity (*I*^2^ = 7.3%) (Supplementary Figure 4A). In the LUM + IVA subgroup and the TEZ + IVA subgroup, the pooled result of FE1 was 52.6 μg/g (95% CI, 23.51–81.69) and 2.50 (95% CI, –1.60 to 6.59), respectively (Supplementary Figure 4A). As for the IRT concentration, the pooled result in the IVA group and the LUM + IVA group was –130.86 ng/g (95% CI, –196.01 to –65.71) and –130.20 ng/g (95% CI, –190.91 to –69.49), respectively (Supplementary Figure 4B), which were almost the same. In the TEZ + IVA group, the pooled result was –61.45 ng/g (95% CI, –89.26 to –33.64) (Supplementary Figure 4B).

### Adverse Events for Single-Arm Studies and Randomized Controlled Trials

In single-arm studies, the rate of any adverse event was almost the same across the studies, ranging from 92.9 to 100% (Table 3). The rate of serious adverse events was different among the studies, ranging from 1.5 to 33.3% (Table 3). The rate of interruption of treatment due to an adverse event varied obviously (from 0 to 11.8%); however, the rate of discontinuation of treatment due to an adverse event was similar among most studies (Table 3). The most common adverse events were cough (from 35.7 to 73.7%), vomiting (from 10.0 to 39.4%), nasal congestion (from 14.3 to 26.5%), rhinorrhea (from 10.0 to 31.6%), and pyrexia (from 10.3 to 39.4%%) (Table 3). The rates of ALT or AST > 5 × upper limit of normal in most of the studies were more than 5% (Table 3).

**TABLE 3:** Adverse events for single-arm studies.

Adverse event	Davies et al. (13)	Milla et al. (14)	Rosenfeld et al. (15)	McNamara et al. (16)	Rosenfeld et al. (17)	Walker et al. (18)	Davies et al. (19)	Zemanick et al. (20)	Sawicki et al. (21)
	
	number of patients (percent)								
	
	IVA (*n* = 34)	LUM + IVA (*n* = 57)	IVA (*n* = 19)	LUM + IVA (*n* = 60)	IVA (*n* = 33)	TEZ + IVA (*n* = 70)	IVA (*n* = 17)	ELX + TEZ + IVA (*n* = 64)	TEZ + IVA (*n* = 130)
Any adverse event	NA	55 (94.8)	18 (94⋅7)	59 (98)	33 (100)	65 (92.9)	16 (94.1)	65 (98.5)	129 (99.2)
Serious adverse event	7 (20.6)	4 (6.9)	4 (21.2)	4 (7)	11 (33.3)	6 (8.6)	NA	1 (1.5)	31 (23.8)
Interruption of treatment due to an adverse event	4 (11.8)	6 (10.3)	0	3 (5)	5 (15.2)	4 (5.7)	0	1 (1.5)	9 (6.9)
Discontinuation of treatment due to an adverse event	1 (2.9)	2 (3.4)	0	3 (5)	5 (15.2)	1 (1.4)	0	1 (1.5)	5 (3.8)
Headache	NA	12 (20.7)	NA	NA	NA	NA	NA	16 (24.2)	20 (15.4)
Cough	19 (55⋅9)	29 (50.0)	14 (73⋅7)	38 (63)	24 (72.7)	25 (35.7)	10 (58.8)	28 (42.4)	73 (56.2)
Vomiting	10 (29⋅4)	6 (10.3)	3 (15⋅8)	17 (28)	13 (39.4)	7 (10.0)	4 (23.5)	7 (10.6)	21 (16.2)
Nasal congestion	9 (26⋅5)	12 (20.7)	NA	10 (17)	7 (21.2)	10 (14.3)	4 (23.5)	10 (15.2)	24 (18.5)
Upper respiratory tract infection	8 (23⋅5)	NA	4 (21⋅1)	10 (17)	5 (15.2)	NA	3 (17.6)	11 (16.7)	31 (23.8)
Rhinorrhea	7 (20⋅6)	NA	6 (31⋅6)	15 (25)	6 (18.2)	7 (10.0)	5 (29.4)	8 (12.1)	NA
Pyrexia	6 (17⋅6)	6 (10.3)	7 (36⋅8)	17 (28)	13 (39.4)	13 (18.6)	5 (29.4)	14 (21.2)	26 (20.0)
Infective pulmonary exacerbation of CF	5 (14⋅7)	12 (20.7)	NA	NA	10 (30.3)	16 (22.9)	NA	NA	60 (46.2)
Constipation	4 (11⋅8)	NA	3 (15⋅8)	7 (12)	NA	1 (1.4)	2 (11.8)	NA	NA
Rash	4 (11⋅8)	NA	NA	NA	4 (12.1)	NA	NA	8 (12.1)	NA
Otitis media	3 (8⋅8)	NA	4 (21⋅1)	7 (12)	6 (18.2)	NA	3 (17.6)	NA	NA
Productive cough	3 (8⋅8)	NA	NA	NA	NA	NA	NA	NA	22 (16.9)
Sinusitis	3 (8⋅8)	NA	2 (10⋅5)	NA	5 (15.2)	1 (1.4)	NA	NA	NA
ALT or AST > 3 × ULN	NA	11 (19.3)	5 (27.8)	9 (15)	10 (30.3)	7 (10)	NA	NA	14 (10.7)
3 × ULN < ALT/AST < 5 × ULN	NA	6 (10.5)	3 (15.8)	NA	1 (3.0)	4 (5.7)	1 (5.9)	NA	6 (4.6)
5 × ULN < ALT/AST < 8 × ULN	NA	2 (3.5)	NA	NA	4 (12.1)	2 (2.9)	NA	NA	6 (4.6)
ALT or AST > 8 × ULN	5 (14.7)	3 (5.3)	2 (11.1)	NA	5 (15.2)	1 (1.4)	NA	NA	2 (1.5)
Abdominal pain	NA	6 (10.3)	NA	NA	5 (15.2)	10 (14.3)	NA	8 (12.1)	21 (16.2)
Cataracts	0	1 (1.8)	0	0	NA	NA	0	NA	NA

In RCTs, the rates of any adverse event and serious adverse events were almost the same between the LUM + IVA group and the placebo group (Table 4). The most common adverse events were cough, pulmonary exacerbation, oropharyngeal pain, headache, pyrexia, upper abdominal pain, upper respiratory tract infection, nasal congestion, and abdominal pain, which were almost even between the treatment group and the placebo group (Table 4).

**TABLE 4:** Adverse events for RCT studies.

Adverse event	Davies, 2013		Ratjen, 2017		Davies, 2021	
	Number of patients (percent)					
	IVA (*n* = 26)	Placebo (*n* = 26)	LUM+IVA (*n* = 103)	Placebo (*n* = 101)	TEZ+IVA (*n* = 54)	Placebo (*n* = 10)
Any adverse event	NA	NA	98 (95)	98 (97)	41(75.9)	NA
Serious adverse event	NA	NA	13(13)	11(11)	2(3.7)	NA
Oropharyngeal pain	NA	NA	15(15)	10(10)	NA	NA
Headache	4 (15)	7 (27)	13 (13)	9 (9)	8(14.8)	NA
Cough	19 (73)	13 (50)	46 (45)	47 (47)	8(14.8)	NA
Vomiting	7 (27)	2 (8)	10 (10)	10 (10)	4 (7.4)	NA
Nasal congestion	4 (15)	5 (19)	17 (17)	8 (8)	3 (5.6)	NA
Upper respiratory tract infection	2 (8)	6 (23)	13 (13)	10 (10)	NA	NA
Rhinorrhoea	4 (15)	3 (12)	10 (10)	5 (5)	3 (5.6)	NA
Pyrexia	7 (27)	6 (23)	15 (15)	20 (20)	NA	NA
Infective pulmonary exacerbation of CF	8 (31)	8 (31)	20 (19)	18 (18)	NA	NA
Constipation	3 (12)	2 (8)	NA	NA	NA	NA
Rash	3 (12)	2 (8)	NA	NA	NA	NA
Otitis media	1 (4)	4 (15)	NA	NA	NA	NA
Productive cough	5 (19)	2 (8)	18 (17)	6 (6)	7 (13)	NA
Sinusitis	3 (12)	2 (8)	NA	NA	NA	NA
ALT/AST > 3 × ULN	NA	NA	13(13)	8 (8)	NA	NA
3 × ULN < ALT/AST < 5 × ULN	NA	NA	NA	NA	3 (5.6)	2 (20)
Abdominal pain	3 (12)	4 (15)	10 (10)	10 (10)	3 (5.6)	NA
						

## Discussion

Cystic fibrosis (CF) begins early in life, with injury to the pancreas beginning *in utero* (25), and lung disease can appear at birth and progress throughout childhood (26). Hence, early treatment using a CFTR modulator in life is expected to prevent the development of end-organ damage. The physiological status of children is different from that of adults, and hence the effectiveness and safety of the CFTR modulator need to be explored.

Ivacaftor (IVA) was first conducted in double-blind RCTs in patients older than 12 years (STRIVE) (7) and children aged 6–11 years (ENVISION) (22), which demonstrated a significant improvement in ppFEV_1_, reduction in SwCI, and weight gain. Hence, IVA was approved for treating patients with CF having CFTR gating mutation. The first-generation CFTR correctors LUM and TEZ (which were designed on the basis of LUM) were used to repair aberrant assembly of the full-length proteins. However, p.Phe508del mutation led to not only a processing and trafficking defect but also premature degradation and defects in the gating and stability for CFTR. This was the reason why LUM or TEZ monotherapy was not effective in patients with F/F mutation (27). Hence, dual combination treatment, which includes IVA (LUM + IVA or TEZ + IVA), should be used for patients with F/F or F/RF mutation.

In the meta-analysis of the included RCTs, the ppFEV_1_ in the treatment group was significantly increased compared with that in the placebo group. Simultaneously, the heterogeneity was significant among the studies due to different drugs and genotype mutations. The ppFEV_1_ in the studies by Ratjen et al. (23) and Davies et al. (24) remained stable in the treatment group but declined in the placebo group. This finding proved that lung function progressively declined in patients with CF; hence, early preservation of lung function is important for pediatric patients (23).

The subgroup analysis could not be conducted because of the limited number of RCTs. However, in single-arm studies, the heterogeneity among the included studies moderately decreased, especially in the LUM + IVA subgroup. In the TEZ + IVA group, the children with F/F and F/RF mutations were simultaneously included in the studies, but these two mutations reacted to the drug combination differently, causing heterogeneity. Hence, the CFTR modulator manifested an improvement in ppFEV_1_ for children with gating mutation or at least one p.Phe508del mutation. Evidence suggested that LCI_2.5_ was more sensitive to detect early structural lung abnormalities, particularly in younger children, compared with ppFEV_1_ (28, 29). In this meta-analysis, irrespective of RCTs or single-arm studies, LCI_2.5_ improved. Therefore, intervention with CFTR modulators in children with CF improved lung function and offered the opportunity to slow lung function decline that occurred over time.

Sweat chloride concentration (SwCI) is an established measurement of CFTR function (21). The concentration of SwCI reduced significantly compared with that in the placebo group. The concentration of SwCI was more reduced in the LUM + IVA and TEZ + IVA groups than in the IVA monotherapy group. The reason was perhaps related to different genotype mutations. CFTR gating mutation was more reactive than F/F or F/RF mutation to IVA (27).

The pooled CFQ-R score demonstrated an increase in the treatment group relative to the placebo group. According to the adult version of the CFQ-R score, the minimally clinically important difference was ≥ 4 points (21, 23). Under this criterion, CFTR modulators could increase the life quality of children with CF. However, the child’s version did not have a widely accepted minimal clinically important difference (18). Moreover, the baseline of the CFQ-R score in the included studies was around 80 points, which suggested that the children had a relatively low burden of respiratory symptoms at baseline (21). Hence, the improvement in the CFQ-R score should be interpreted carefully.

The nutritional status is an important consideration in pediatric patients because improvements in growth are associated with better lung function (23, 24). A slow gain in the weight of patients with CF may be attributed to multiple factors, including pancreatic insufficiency leading to intestinal malabsorption, reduced appetite, diabetes, and higher metabolic rate caused by chronic lung inflammation (22). The weight increased in the treatment group compared with the placebo group. However, the BMI and BMI-for-age z-score were not statistically different between the treatment and placebo groups. Similarly, the weight, BMI, and stature were improved under treatment in single-arm studies; however, their associated z-scores were not obviously increased. The potential reason for inconsistent results might be the limited number of patients in the included studies.

FE1 concentration is a measurement for evaluating exocrine pancreatic function. If the FE1 level is < 200 μg/g, it means that exocrine pancreatic function is under insufficiency. The pooled estimates of FE1 increased obviously in the IVA and LUM + IVA group, but the FE1 level of most of the children was still under 200 μg/g after treatment. Another non-specific marker of pancreatic injury is serum IRT, which decreased in the IVA subgroup, LUM + IVA group, and TEZ + IVA group. Although the FE1 level was not over the clinical cutoff level, CFTR modulators might reduce pancreatic inflammation and injury to preserve pancreatic function (24). Additional studies in larger populations are needed to elucidate the precise relationship between FE1 or IRT.

Beyond the prominent benefits of the therapy, the safety was also favorable compared with that of placebo. The adverse events in the therapy groups were nearly the same as those in the placebo groups. Most of the adverse events were mild or moderate; the rate of discontinuation of treatment due to an adverse event in most studies was less than 5%, even in long-term treatment studies (21). The elevated levels of AST or ALT were reported in most of the included studies, which were generally asymptomatic. However, it reminded us that liver function should be assessed before the initiation of CFTR modulator treatment and monitored during treatment. Another particular rare adverse event was cataract, which was found in an animal experiment study (22). One patient developed a cataract of mild severity by Week 24 of the study (14). Therefore, regular optical inspection is needed for children during the treatment.

Only one triple combination therapy (ELZ + TEZ + IVA) has been published, which was approved in the United States to treat CF ≥ 6 years with at least one copy of p.Phe508del mutation (24). The overall manifestations in improving lung function, CFTR protein, CFQ-R score, and nutritional status were obviously significant. The next-generation corrector (ELZ) with a different structure and mechanism of action has been found to increase CFTR processing, trafficking, and function *in vitro* (30, 31). The combination of a next-generation corrector and tezacaftor increased the efficacy of CFTR function to a greater extent than either compound alone (32). However, no RCT result about triple therapy in children is available to date.

This was the first meta-analysis evaluating the effectiveness and safety of CFTR modulators in treating children with CF. The strengths of this meta-analysis were as follows. First, all included single-arm studies were analyzed by the same drug and genotype mutation, thus minimizing bias among the studies as much as possible. Second, the comparison was conducted among RCTs, enhancing the potency of CFTR modulator treatment. Third, similar adverse events were found between the therapy and placebo groups, which demonstrated acceptable safety.

Despite the advantages of CFTR modulator therapy, some limitations of this study should also be considered. First, the number of patients included in the studies was limited, leading to bias in the outcomes. Second, only three RCTs were included, and the treatment of drugs and genotypes were different from each other. Also, the heterogeneity was significant, which made the conclusion less confirmed. Third, the results from the included studies were mostly short term (24 weeks). Hence, additional long-term studies are needed to confirm the results.

In conclusion, CFTR modulator therapy significantly improved or preserved the lung function, CFTR protein function, CFQ-R score, and nutritional status of children with CF using a safe approach. More well-designed RCTs are needed to support the effectiveness and safety, and extend the indications for younger patients diagnosed with CF, to achieve radical treatment for CF before the development of the disease.

## Data Availability Statement

The original contributions presented in this study are included in the article/Supplementary Material, further inquiries can be directed to the corresponding author.

## Author Contributions

QL and JY: study conception and design and data acquisition. SL and XM: analysis and data interpretation. QL: drafting of the manuscript. All authors contributed to the article and approved the submitted version.

## Conflict of Interest

The authors declare that the research was conducted in the absence of any commercial or financial relationships that could be construed as a potential conflict of interest.

## Publisher’s Note

All claims expressed in this article are solely those of the authors and do not necessarily represent those of their affiliated organizations, or those of the publisher, the editors and the reviewers. Any product that may be evaluated in this article, or claim that may be made by its manufacturer, is not guaranteed or endorsed by the publisher.

## Supplementary Material

Supplementary Figure 1 Forest plots of the single-arm studies evaluating the effectiveness of **(A)** change in weight and **(B)** weight-for-age z-score.

Supplementary Figure 2 Forest plots of the single-arm studies evaluating the effectiveness of **(A)** change in BMI and **(B)** BMI-for-age z-score.

Supplementary Figure 3 Forest plots of the single-arm studies evaluating the effectiveness of **(A)** change in stature and **(B)** stature-for-age z-score.

Supplementary Figure 4 Forest plots of the single-arm studies evaluating the effectiveness of **(A)** FE1 concentration and **(B)** IRT concentration.
