# Supplementary material for: Effectiveness and Safety of Cystic Fibrosis Transmembrane Conductance Regulator Modulators in Children With Cystic Fibrosis: A Meta-Analysis
Source: Front Pediatr. 2022 Jun 29;10:937250. doi: 10.3389/fped.2022.937250 (PMC9276987; doi:10.3389/fped.2022.937250)
Supplement: Supplementary file 2 [file Table_2.DOCX]

**Supplementary table 2 Literature search strategy in different database**

| **Database** | **Search strategy** |
| --- | --- |
| **PubMed** | (((("elexacaftor"[Supplementary Concept] OR "elexacaftor"[All Fields] OR "vx445"[All Fields] OR ("elexacaftor"[Supplementary Concept] OR "elexacaftor"[All Fields])) OR ("ivacaftor"[Supplementary Concept] OR "ivacaftor"[All Fields]) OR ("tezacaftor"[Supplementary Concept] OR "tezacaftor"[All Fields])) OR (("triple"[All Fields] OR "triples"[All Fields]) AND ("therapeutics"[MeSH Terms] OR "therapeutics"[All Fields] OR "therapies"[All Fields] OR "therapy"[MeSH Subheading] OR "therapy"[All Fields] OR "therapy s"[All Fields] OR "therapys"[All Fields]))) OR (("vx 659"[Supplementary Concept] OR "vx 659"[All Fields] OR "vx659"[All Fields]) AND ("ivacaftor"[Supplementary Concept] OR "ivacaftor"[All Fields]) AND ("tezacaftor"[Supplementary Concept] OR "tezacaftor"[All Fields])) AND ("cystic fibrosis"[Title/Abstract] OR "CFTR"[All Fields])) AND ("children") |
| **Web of Science** | ((((TI=(elexacaftor)) AND TI=(ivacaftor)) AND TI=(tezacaftor)) OR TI=("CFTR")) AND (TI=("cystic fibrosis") AND (TI=("children")) |
| **Cochrance** | (("cystic fibrosis")):ti,ab,kw AND (("elexacaftor" AND “ivacaftor” AND “tezacaftor” OR "CFTR")):ti,ab,kw AND ("children"):ti,ab,kw |
