# Supplementary figures and images for: Effectiveness and Safety of Cystic Fibrosis Transmembrane Conductance Regulator Modulators in Children With Cystic Fibrosis: A Meta-Analysis
Source: Front Pediatr. 2022 Jun 29;10:937250. doi: 10.3389/fped.2022.937250 (PMC9276987; doi:10.3389/fped.2022.937250)

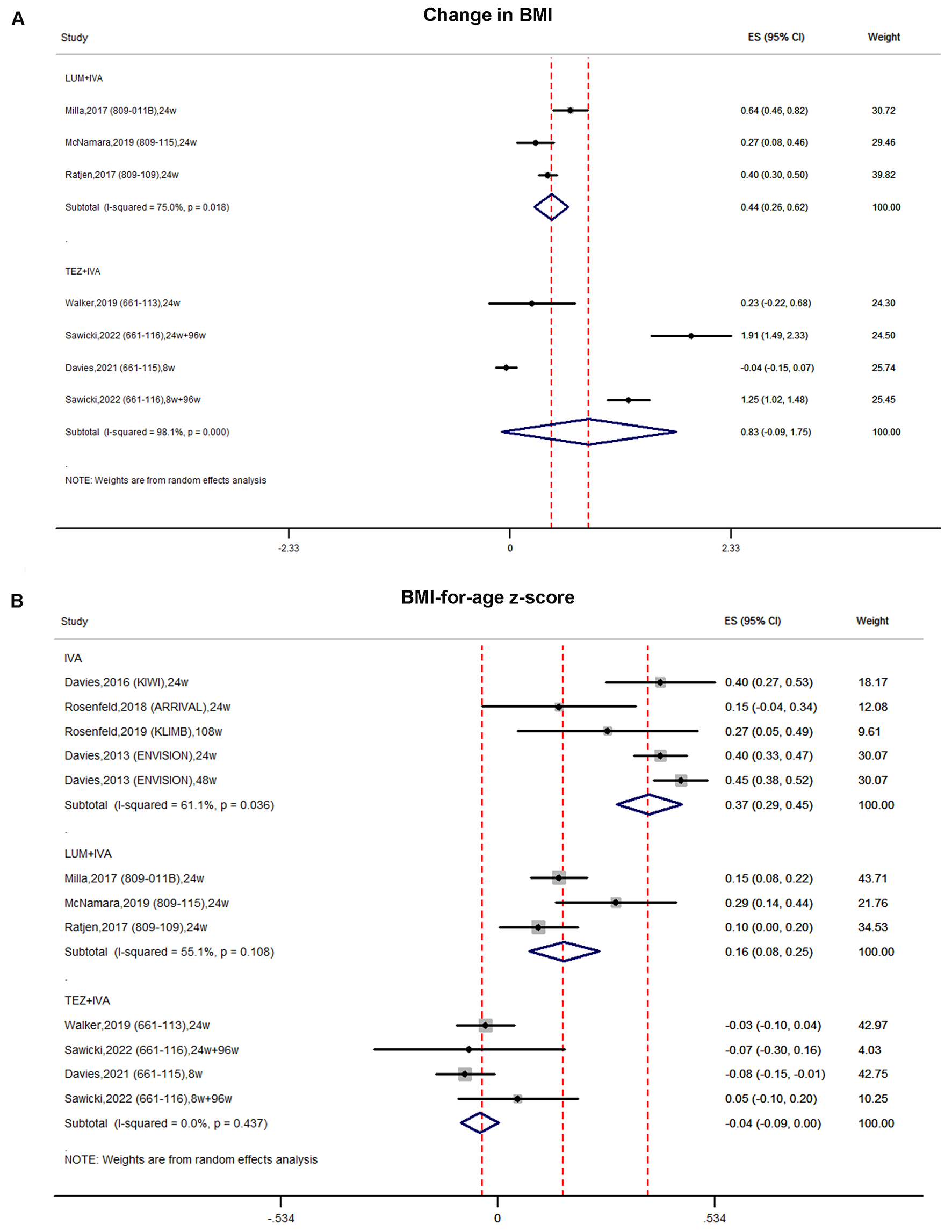

Supplement: Supplementary Figure 1 — Forest plots of the single-arm studies evaluating the effectiveness of (A) change in weight and (B) weight-for-age z-score. [file Image_1.TIF]

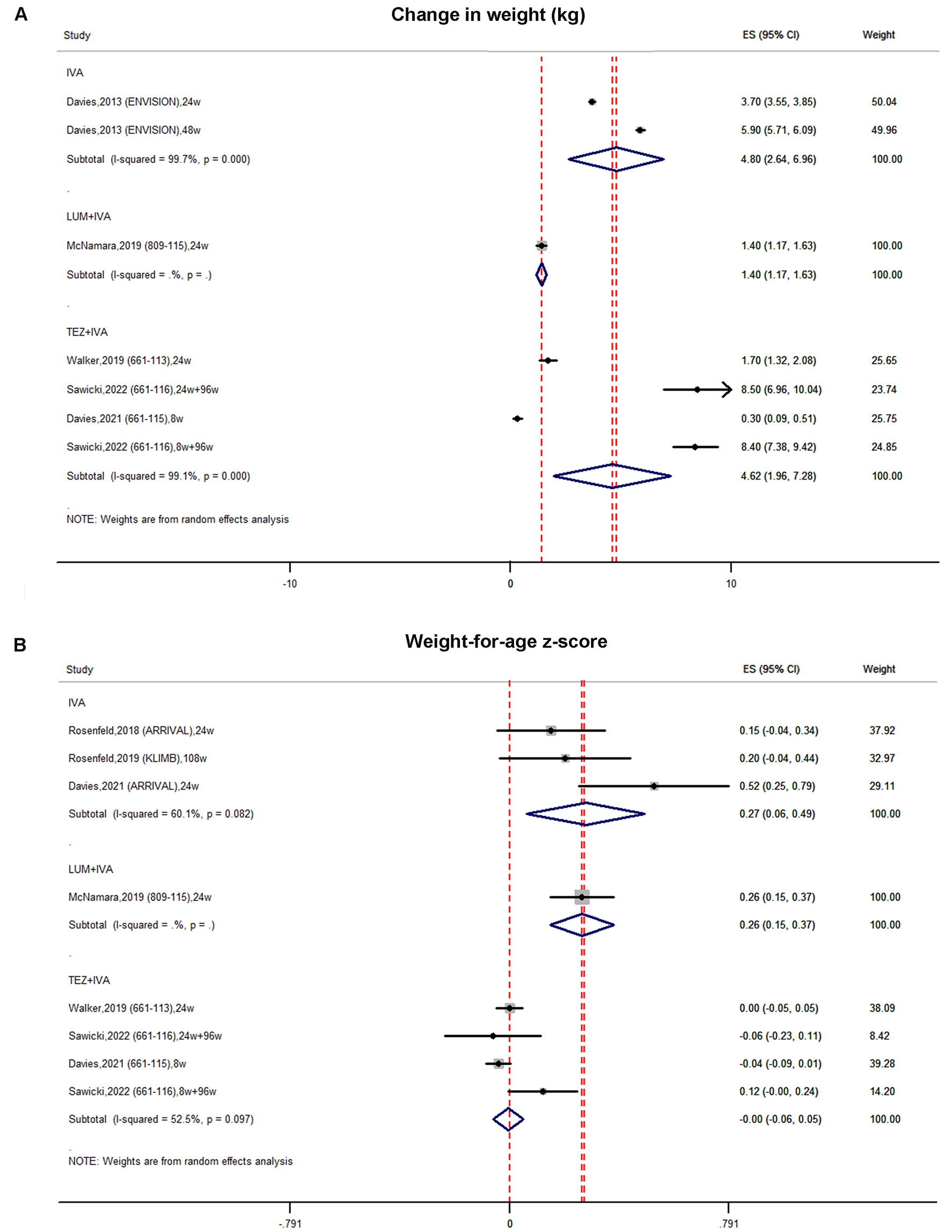

Supplement: Supplementary Figure 2 — Forest plots of the single-arm studies evaluating the effectiveness of (A) change in BMI and (B) BMI-for-age z-score. [file Image_2.TIF]

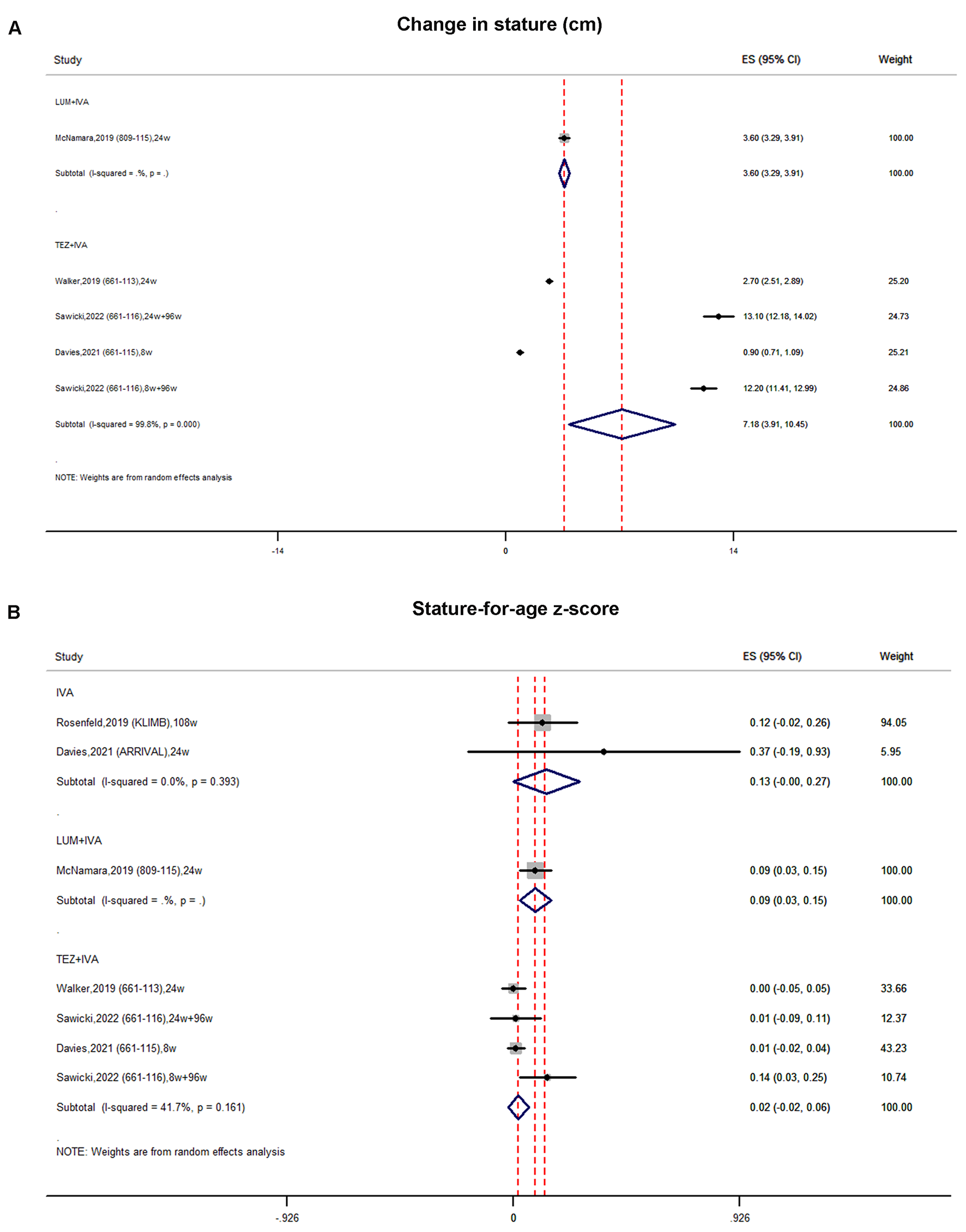

Supplement: Supplementary Figure 3 — Forest plots of the single-arm studies evaluating the effectiveness of (A) change in stature and (B) stature-for-age z-score. [file Image_3.TIF]

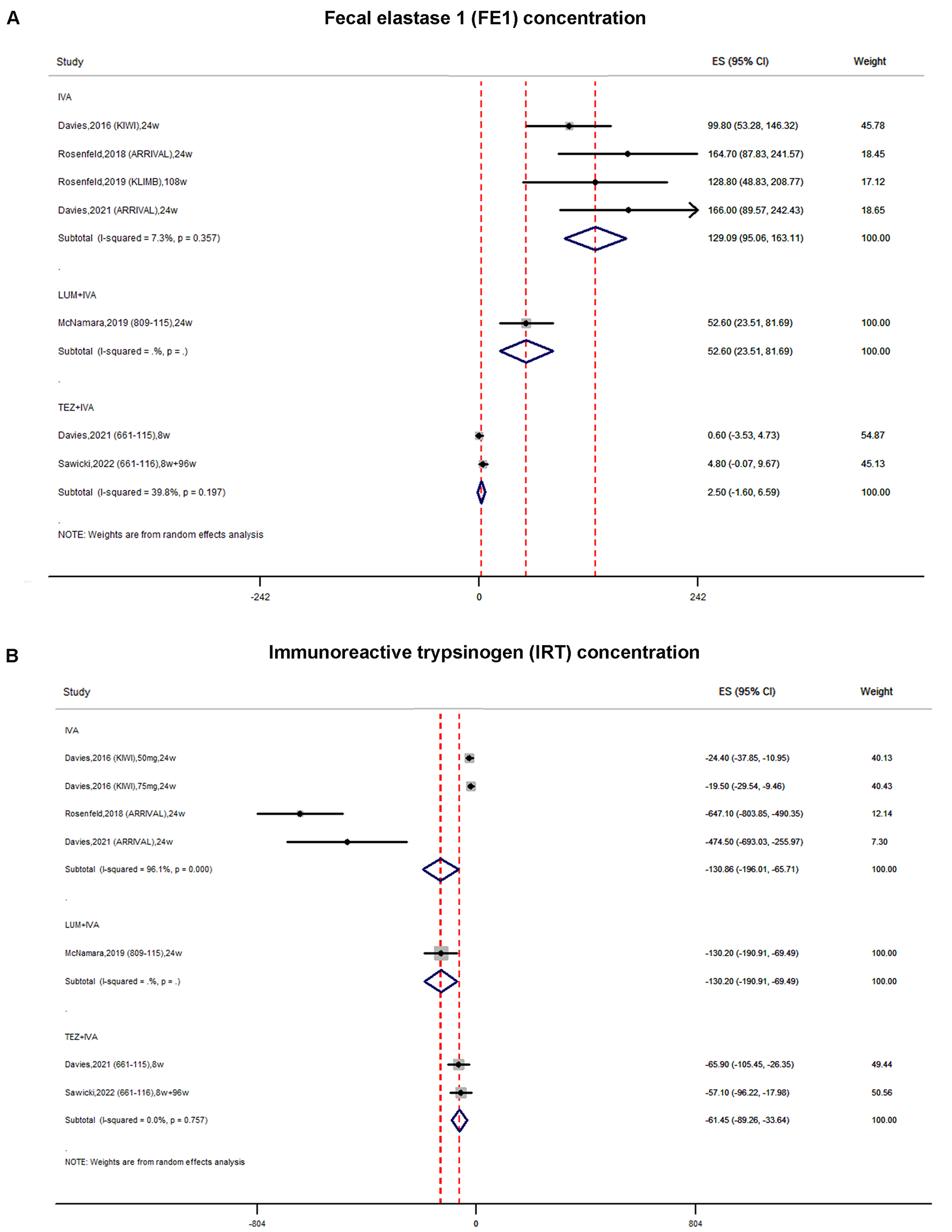

Supplement: Supplementary Figure 4 — Forest plots of the single-arm studies evaluating the effectiveness of (A) FE1 concentration and (B) IRT concentration. [file Image_4.TIF]
